# The Peter Pan paradigm

**DOI:** 10.1186/1742-4682-5-1

**Published:** 2008-01-08

**Authors:** J Craig Cohen, Janet E Larson

**Affiliations:** 1 The Brady Laboratory, Section of Neonatology, Department of Pediatrics, Stony Brook University Medical Center, Stony Brook, NY 11794, USA

## Abstract

Genetic and environmental agents that disrupt organogenesis are numerous and well described. Less well established, however, is the role of delay in the developmental processes that yield functionally immature tissues at birth. Evidence is mounting that organs do not continue to develop postnatally in the context of these organogenesis insults, condemning the patient to utilize under-developed tissues for adult processes. These poorly differentiated organs may appear histologically normal at birth but with age may deteriorate revealing progressive or adult-onset pathology. The genetic and molecular underpinning of the proposed paradigm reveals the need for a comprehensive systems biology approach to evaluate the role of maternal-fetal environment on organogenesis.

You may delay, but time will not

Benjamin Franklin

USA Founding Father

## Background

The fragility of the developmental process is well known with estimates as high as 70% of conceptions lost in early pregnancy [1]. Genetic defects are known to produce numerous changes in both metabolic and morphologic characteristics of the fetus [2]. The choreography of genes necessary for successful fetal development is amply demonstrated by embryonic lethal [3] and other phenotypes [4-8] in knockout mouse models. Thus, genetic defects introduced in the developmental cascade contribute to structural malformations. But organogenesis requires the genetic choreography to occur not just in sequence but also during a sensitive time period. What happens when the genes regulating the developmental time clock [9,10] are mutated or delayed by epigenetic factors? Can the organ continue to develop outside the fetal environment and if not, what are the consequences of retaining fetal characteristics in an adult?

The best model for addressing these questions related to organogenesis and timing is the premature infant. Modern neonatology has extended viability to 23 weeks in the 40 week human gestation, but what are the consequences of completing less than 60% of gestation on the human fetus? The obvious immediate consequences seen in any neonatal unit include poor lung function, intestinal deficiencies, immune system deficiencies, and poor renal function. These immediate problems combined with therapies used to combat them can lead to significant morbidity and mortality in the premature infant.

Infants surviving the immediate organ deficiencies and therapies of premature birth, however, do not grow into healthy adult. Rather, numerous epidemiological and clinical observations have shown that prematurity is associated with many adult onset diseases (Table 1). The epidemiologic basis for late or adult-onset diseases, following either premature birth or low birth weight, includes poor pre-natal maternal nutrition and smoking during pregnancy both of which are reviewed previously [11,12]. But these epidemiologic correlations do no provide a theoretical, molecular basis for explaining late onset of diseases related to delays in organogenesis.

**Table 1:** Late-onset clinical presentations associated with premature birth.

Clinical Presentation	**References**
Diabetes	[27, 28, 62, 63]
Inflammatory Bowel Diseases	[64]
Obesity	[12, 63]
Cardiovascular Disease	[28]
Asthma	[65]
Sleep Disordered breathing	[66]
Sudden Infant Death Syndrome (SIDS)	[13, 67]

Conceptualizing the effect of small developmental disturbances on solid organs such as the lung, liver, intestines and kidneys can be difficult because morphological differences may be minor. If one considers an organ with functional characteristics that are morphologic, however, then the concept of developmental disruption becomes obvious. A good example would be the hand. Numerous examples of impaired development through either genetic defects or blood flow interruption result in obvious deformities including webbing, polydactyl and complete and partial absences of digits. Visualization of changes in solid organ development is less obvious but the effects on function have the same impact as a hand without a thumb.

**Figure 1:**
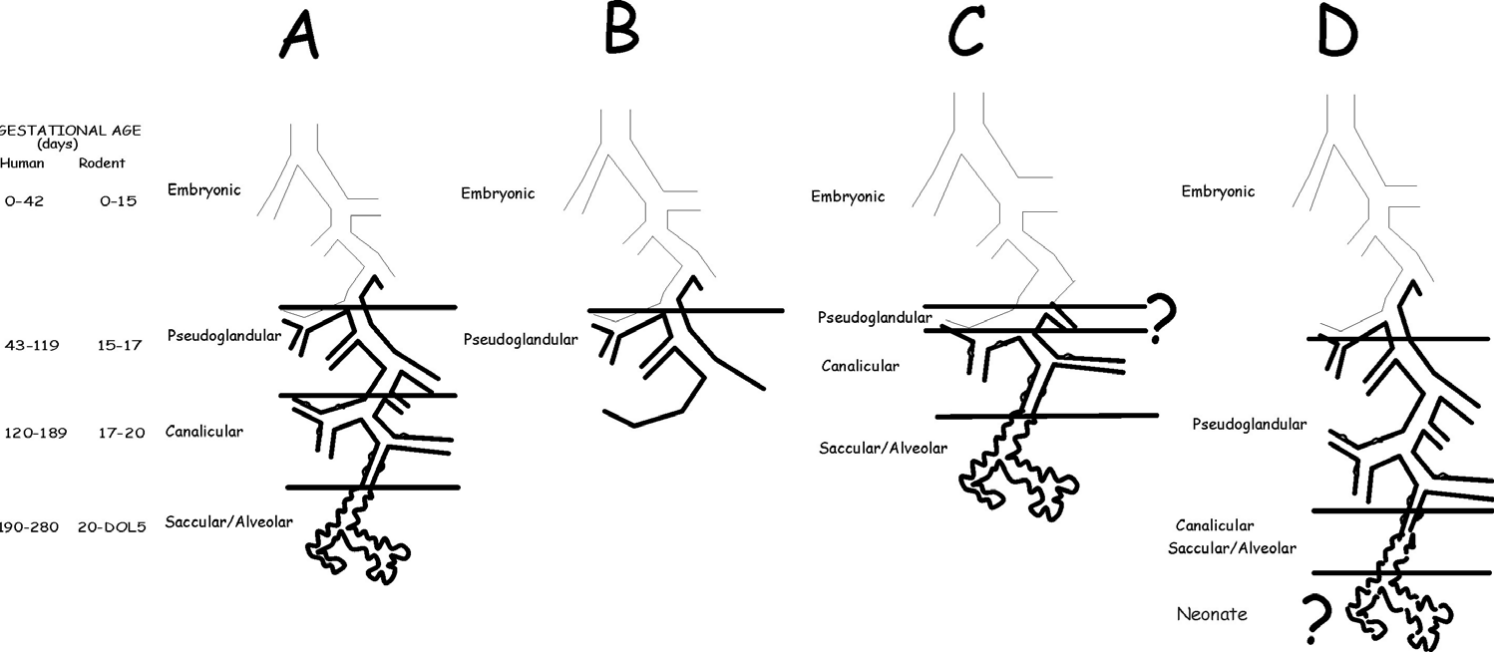
**Lung development and the effects of developmental disruption**. A-Normal lung differentiation; B-Disrupted growth; C-Premature transition resulting in abbreviated pseudoglandular stage; D-Delayed transition leading to incomplete canalicular and saccular/alveolar stages.

As an example, lung growth and development (Fig. 1A), like the hand, is precisely timed throughout gestation [13]. In humans functional respiratory structures are complete by birth although alveolarization continues until an adult number of alveoli are reached at 2–3 years of age; however, in the mouse and rat functional alveolarization is completed after birth by day of life (DOL) 5. The embryonic human lung can be identified at 28 days with trachea and bronchi expanding from the lung bud. During the pseudoglandular stage further branching occurs and the airways are lined with multipotent stem cells. Branches of terminal bronchioles are formed and respiratory bronchioles begin as out pockets. These pockets of epithelial cells expand during the saccular and alveolar stages to form terminal alveoli which are necessary for gas exchange. Normal lung organogenesis is a carefully programmed process that can be disrupted and result in abnormal lung structure and function.

Three types of disruptions can occur in lung organogenesis (Fig. 1B–D). The most obvious are genetic mutations that completely block lung growth (Fig. 1B) resulting in a non-functional lung and fetal or neonatal demise [14-16]. The less obvious yet equally important disruptions to normal lung function are those that impair or impede specific events in organogenesis. A genetic mutation or environmental insult to the fetus through the mother can result in either a premature (Fig. 1C) or prolonged (Fig. 1D) transition to a later stage of development. In the former case, structures, which are normally propagated, are lost along with their function. In the latter case, slower growth results in late development of important structures and functions prior to normal birth. In both cases, the histologic structure of the lung may grossly appear normal; however, the actual functional equivalency of the cells may be significantly altered.

**Figure 2:**
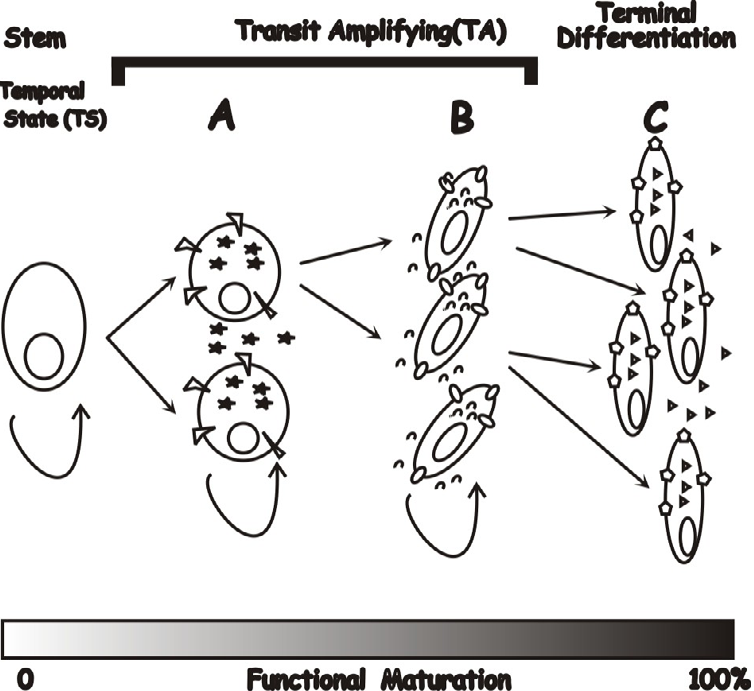
**Stem cell differentiation during organogenesis**. Progression through varying transit amplifying (TA) cell stages of functional maturity (bottom gray scale) called temporal states (TS) A-C. Various geometric shapes (stars, ovals, triangles, etc) represent either intracellular, extracellular, or membrane associate markers of differentiation.

Two questions arise from these models. First, can discontinuity between stages in a developmental cascade alter the cell type or their function? During each stage of organogenesis, stem cells are progressing through stages of transit amplifying (TA) cells in which the functional characteristics of each generation is maturing to that of a terminally differentiated cell type (Fig. 2). During organogenesis the environment progresses through different temporal states (TS) in which the TA cells produce products necessary for differentiation that are not necessary needed for final functionality. As an example, in TS-A (Fig. 2) may produce products that are required for development of specialized structures. Proteins produced in TS-B, however, may induce rapid cell growth and increased tissue complexity. If this process is abbreviated as proposed in Fig. 1C, then the terminal differentiation state can not be reached and downstream functional characteristics of the cell will be missing.

Secondly, when there is a delay in progression (Fig. 1D), does normal organogenesis continue postnatally? Because the fetal environment plays a significant role in differentiation, preterm birth arrests organ development with retention of fetal characteristics. Thus, all or part of the organ would remain in a fetal temporal state (e.g. TS-B; Fig. 2), significantly different from that of the terminally differentiated tissue (TS-C, Fig. 2).

**Figure 3:**
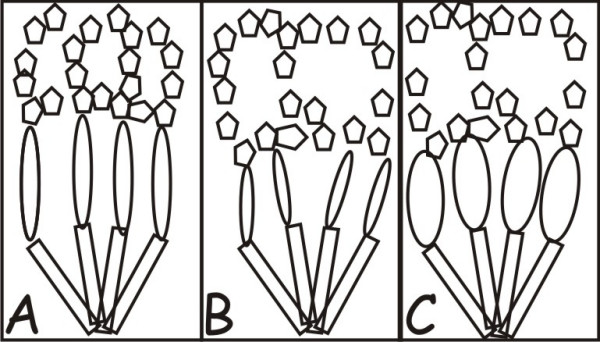
**Architecture and developmental programming**. Normal architecture depends on building layers that are based on both quantity and timing of components with an overall structural size limitation (Panel A). Premature activation of subsequent stages disrupts primary base structure requiring downstream components to fill a space larger than normally required resulting in less complexity (Panel B). Likewise, prolonging production of the upstream components increases their complexity at the expense of downstream structures (Panel C), resulting in a hypoplastic structure similar to that seen in disrupted growth of upstream components.

Delay and disruption of organogenesis could result in two different phenotypes because the immature tissue would be missing different functional gene sets. Distinguishing these two would be dependent upon a developmental gene expression knowledge base. However, it is possible to get similar morphologic phenotypes but different physiology. As illustrated in Figure 3, if one takes a case in which a protein is required for triggering the normal final stage of complex organ architecture (Fig. 3A), disrupting the stage that precedes this protein could result in its premature activation without sufficient support structure (Fig. 3B), resulting in a hypoplastic structure. Likewise, prolonging the preceding stage by delaying activation of these proteins could lead to some gain in complexity in the supporting structure. However, similar decreased structural complexity of this final stage would result (Fig. 3C) due to a decrease in the time interval between activation of terminal differentiation and birth. Morphologically both tissues would appear hypoplasia of the terminal structure; because the balance among structural components is altered the physiology could be quite different. Thus, distinctions between delayed and disrupted organogenesis will be dependent upon biochemical makers of differentiation, structural characteristics of the tissue, and perhaps most importantly the physiologic functions of the modified organ.

There are numerous examples of failed differentiation due to specific gene defects leading to deficient cell types. In the lung, mutations in lamellar body formation lead to structural changes leading to chronic inflammatory diseases [17-19]. Mutation of Arnt, a gene required for hepatic vascularization, results in a persistent embryonic liver phenotype that demonstrate decreased survival and changes in fat and carbohydrate metabolism after birth [20]. Bapx1 is required for separation of the pancreas and spleen into distinct organs [21]. In each of these single gene defects, slow or inhibited organogenesis results in a suboptimal functioning organ and a subsequent pathology.

The physical environment of the fetus can also impact the dynamics of organogenesis. One of the more common prototypes is congenital diaphragmatic hernias which result in hypoplastic lung disease. Surgical blocking of the trachea [22] or *in utero *cystic fibrosis transmembrane conductance regulator (CFTR) gene therapy [23] to promote stretch induced lung differentiation can reverse the effect, but postnatally this condition has a poor prognosis with high morbidity and mortality. Likewise, amniotic fluid blockage to the intestines alters villus formation and function [24].

Probably the least understood and most subtle effectors of developmental delay are fetal environmental insults. Maternal smoking, alcohol consumption, diabetes, and diehylstilbesterol use have all been linked to significant long term consequences on the fetus [11,19,25-28].

## Discussion

What are diseases of organogenesis that result from developmental delay and not disruption called? In premature infants terms like fetal and immature are used to describe their gestational correct, underdeveloped organs. We propose that the temporal state of many tissues can become fixed at birth, resulting in organs that are functionally immature. Thus, we use the term Peter Pan Paradigm for diseases that occur due to developmental delay or disruption resulting in a seemingly histological, normal organ with functional immaturity. Developmental delay produces tissues that at birth are in a gestational abnormal temporal state, either fetal (e.g. TS-B; Fig. 2) or hybrid (e.g. TS-B/C). Thus, like the fictional namesake, these affected organs never fully mature and remain at a nexus between the fetal and mature temporal states.

Changes in the organs trapped in either a complete or partial fetal temporal state can be varied. As illustrated in Fig. 2 receptors and secreted products may be different. In addition, functions such as regulated secretion could be affected. This functional immaturity results in progressive incapacitation of the organ with associated late-onset disease manifestations. Possible syndromes included in this category would be any progressive or late-onset disease that exhibited multi-factorial genetics. Obviously, these diseases would present with very heterogeneous phenotypes because the more severe the delay, the earlier the average temporal state of the tissue.

The prototypical disease in the Peter Pan paradigm is Cystic Fibrosis (CF). The natural history of this disease includes progressive multi-system failure. There is a lack of phenotype genotype correlation suggesting the role of multiple genes or environment [29,30]. CF is a recessive disorder yet the human phenotype can occur in the absence of homozygous mutations in the CFTR gene [31] and the heterozygous knockout mice have a unique lung phenotype [32]. Expression during embryonic lung growth is associated with genes required for normal development [3,10,33]. CFTR expression occurs at high levels in the developing lung [34] and other tissues [35]. Furthermore, transient *in utero *replacement to increase or decrease expression of CFTR in animal models alters the lung and intestinal CF phenotype [23,36-43]. Thus, there are multiple independent lines of evidence suggesting a role of CFTR in normal organogenesis of the lung, intestines, and other organs.

Born with more or less normal histology of the lung, pancreas, and liver, but with a more serious intestinal phenotype, CF patients exhibit progressive lung disease largely blamed on decreased mucous hydration [44,45]. However, the hypothesis that the altered water balance and resultant inflammatory lung disease is due solely to lack of CFTR in the post-natal lung is contradicted by both the CF phenotype in the absence of CFTR mutations [31] and the finding of CF-like lung disease following transient *in utero *knockout of CFTR [38]. Thus, the lung disease is consistent with the presence of under differentiated cells that maintain a functionally immature phenotype in a structure; that initially exhibits normal histology; and that contribute to the altered mucous phenotype. Environmental challenges to the lungs as they age result in increased infections and the bronchiectasis. A similar process underpins CF-related diabetes [46,47] and liver function decreases with time resulting in disease [48].

As previously described regarding progression of development from one stage to another (Fig. 1), the recent finding that CFTR influences stretch-induced differentiation [37] further delineates CF as a Peter Pan disease. Stretch induced differentiation is essential for normal lung development [49-52]. Amniotic fluid flow is also essential for normal gut differentiation [24]. Thus, the major organs involved in the CF phenotype are all dependent upon amniotic fluid flow and mechanochemical regulated differentiation [51,53]. In the total absence of CFTR this flow would be dependent upon other, less effective, mechanisms to drive development such as rho kinase [54] and fetal breathing [55]. In addition, environmental exposure of the mother to nicotine or other agents could affect the final phenotype by affecting muscle contractions necessary for stretch induction [25,56,57]. Thus, the lack of phenotype-genotype correlation and a common mechanochemical origin would account for the variable effects seen in multiple organ systems.

The intestine may be one of the more susceptible organs for Peter Pan diseases. As discussed earlier inflammatory bowel diseases are linked epidemiologically with prematurity (Table 1). Thus, delay in intestinal development could alter cell maturation and the metabolic balance of the intestines. One of the most common Peter Pan diseases may prove to be obesity and diabetes. The ability to maintain metabolic balance depends on developmental and transcriptional control systems of the intestinal epithelium [58,59]. Thus, interruption or delay leading to under differentiation of intestinal epithelium would have a significant impact on metabolic activity. In what may be considered an early prelude to Peter Pan Paradigm, a relationship between metabolic balance and fetal development was originally proposed in the "Thrifty Phenotype Hypothesis"[60].

Lung diseases such as asthma, allergies, and sudden infant death syndrome (SIDS) may be related to delay in development. All of these conditions are associated with prematurity (Table 1) and from Fig. 1, one can readily visualize how slowing progress in differentiation of succeeding structures would have a cascading effect on lung function. The central role of stretch-induced differentiation to lung development [37,51,53,54] strongly suggests that deficiencies in amniotic fluid volume, depression of fetal breathing [55], or inhibition of smooth muscle contractions [49,52] with agents such as nicotine [57] could depress and delay differentiation of the bronchioles leading to under differentiated cell types and airways. In fact this is the case as previous studies showed persistence of fetal gene expression and functional immature phenotype in allergic asthma [61].

## Competing interests

The author(s) declare that they have no competing interests.

## Authors' contributions

JCC and JEL developed this concept jointly.
